# Trajectory Following Control of an Unmanned Vehicle for Marine Environment Sensing

**DOI:** 10.3390/s24041262

**Published:** 2024-02-16

**Authors:** Tegen Eyasu Derbew, Nak Yong Ko, Sung Hyun You

**Affiliations:** 1 Interdisciplinary Program in IT-Bio Convergence Systems, Department of Electronic Engineering, Chosun University, Gwangju 61452, Republic of Korea; eyasuderbew2008@gmail.com; 2 Department of Electronic Engineering, Chosun University, Gwangju 61452, Republic of Korea; you@chosun.ac.kr

**Keywords:** sensing, marine, vehicle, sliding mode control, trajectory following, estimation, Lyapunov stability, genetic algorithm, chattering

## Abstract

An autonomous surface vehicle is indispensable for sensing of marine environments owing to its challenging and dynamic conditions. To accomplish this task, the vehicle has to navigate through a desired trajectory. However, due to the complexity and dynamic nature of a marine environment affected by factors such as ocean currents, waves, and wind, a robust controller is of paramount importance for maintaining the vehicle along the desired trajectory by minimizing the trajectory error. To this end, in this study, we propose a robust discrete-time super-twisting second-order sliding mode controller (DSTA). Besides, this control method effectively suppresses the chattering effect. To start with, the vehicle’s model is discretized using an integral approximation with nonlinear terms including environmental disturbances treated as perturbation terms. Then, the perturbation is estimated using a time delay estimator (TDE), which further enhances the robustness of the proposed method and allows us to choose smaller controller gains. Moreover, we employ a genetic algorithm (GA) to tune the controller gains based on a quadratic cost function that considers the tracking error and control energy. The stability of the proposed sliding mode controller (SMC) is rigorously demonstrated using a Lyapunov approach. The controller is implemented using the Simulink^®^ software. Finally, a conventional discrete-time SMC based on the reaching law (DSMR) and a heuristically tuned DSTA controller are used as benchmarks to compare the tracking accuracy and chattering attenuation capability of the proposed GA based DSTA (GA-DSTA). Simulation results are presented both with or without external disturbances. The simulation results demonstrate that the proposed controller drives the vehicle along the desired trajectory successfully and outperforms the other two controllers.

## 1. Introduction

Marine environment sensing, monitoring, and management are essential to protect the ocean. In this regard, autonomous surface vehicles (ASVs) or unmanned surface vehicles (USVs) (hereafter, ASVs or USVs can be used interchangeably) play a crucial role in sensing and monitoring the ocean, providing essential information [1,2,3,4]. The ocean covers two-thirds of the Earth’s surface and has a substantial impact on weather patterns, marine ecosystems, coastal regions, and the dynamics of climate change [1]. It is a source of energy, food, and materials; moreover, it is used for transportation and recreational purposes [5]. Marine ecosystem degradation, ocean temperature rise, plastic waste contamination, and pollution, etc are posing a threat to mankind [1]. To address this problem, surface vehicles equipped with sensors can be applied to collect data. For autonomous, efficient, and accurate sensing of marine environments, following a desired sensing trajectory is essential. Moreover, autonomous trajectory following of USVs has been utilized in various application areas, including military operations, transportation, surveillance, and offshore infrastructure [6,7,8,9]. This paper focuses on the trajectory following control of USVs.

**Figure 1:**
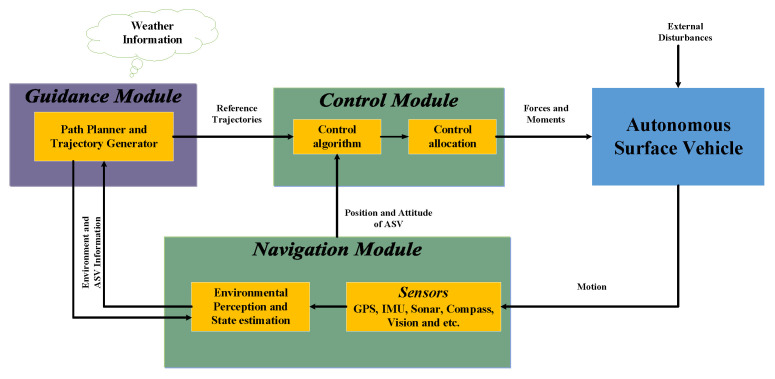
Guidance, navigation and control (GNC) block diagram.

Different types of USVs are used depending on the application area; however, all USVs share some basic components, such as the hull, data collection equipment, communication systems, guidance, navigation and control (GNC), and propulsion and power systems [10]. The GNC system plays a significant role in a vehicle’s capability of accomplishing a given mission. One of the main challenges to develop USVs is the lack of a reliable and automated GNC that can operate in sophisticated and hazardous operating conditions, and overcome the possible failures in sensors, actuators, and communication [10,11]. The general block diagram of GNC is shown in Figure 1. In the navigation system, different onboard sensors, such as global positioning system (GPS), inertial measurement unit (IMU), compass, sonars, and cameras can be incorporated to measure the states of the vehicle, that is, position, orientation, velocity, and acceleration, as well as to gather data regarding the environment for scientific research. Additionally, state estimator or observer can be used to estimate the states of the vehicle if there are no adequate sensors or to estimate the unmeasurable states of the system.

The guidance system generates a continuous and smooth trajectory to the control system based on the information provided by the navigation system, desired mission, and environmental conditions. In this study, we focus on developing the control system as part of the GNC, assuming that the navigation system provides the necessary states, which are the vehicle’s position, orientation, velocity, and acceleration. The control system operates to drive the vehicle to follow the command velocity provided by the guidance module, utilizing information provided by the navigation systems and minimizing the position trajectory error [10]. Therefore, the tracking error in the research indicates the difference between the controlled velocity and the commanded velocity. The tracking error in the position is considered by the guidance system.

Trajectory following for surface vehicles is challenging, owing to the complexity and nonlinearity of vehicle models and various environmental disturbances such as wind, waves, and currents [12]. To this end, researchers have employed various control strategies. One of the most widely used controllers is the proportional, integral, and derivative (PID) controller [13,14,15,16], but it lacks robustness and is highly dependent on the accuracy of the system dynamics [17]. Model predictive control (MPC) was applied in [18,19], which is sufficient for handling system constraints; but it is computationally intensive when the prediction horizon increases, making practical implementation difficult [12]. Other control methods that have been applied include linear quadratic regulator (LQR) [20,21,22], feedback linearization [21,23], backstepping control [24,25], fuzzy logic and its variants [26], neural networks [27], adaptive control [28] and other well-known control algorithm commonly used for trajectory-following applications is sliding mode controller [24].

Sliding mode control (SMC) is a robust control strategy that ensures insensitivity to matched uncertainties in line with the control input. This allows the straightforward implementation of a robust controller, provided the uncertainties are bounded [29,30]. The fundamental principle of the SMC is to select a sliding surface within the state space in which the system has the desired characteristics. Subsequently, a controller can be designed to force the system trajectories from any initial condition within the state space to the sliding surface within finite time. Additionally, the trajectories can be maintained on the surface afterwards. In essence, SMC comprises two parts: equivalent control responsible for bringing the state trajectories from any initial condition to the sliding manifold, and discontinuous control that keeps the trajectories on the surface after they are brought to the manifold. Thus, the system is insensitive to model uncertainty and bounded exogenous disturbances [29].

However, SMC does not preserve the theoretical sliding motion formulation; rather, it switches to an infinitely high frequency for the sliding manifold. This high-frequency switching is not practically feasible due to the limitations of the actuator bandwidth. Hence, an ideal sliding mode cannot be achieved. Additionally, high-frequency switching of the control signal results in a phenomenon known as the chattering effect. Chattering can result in wear and tear of mechanical actuators as well as high energy consumption. The possible reasons for chattering, according to [30,31], are: (i) even though high-frequency switching is achievable, the existence of “parasitic dynamics“, that is, unmodeled actuator and sensor dynamics, in series with the system could result in a small oscillation around the sliding manifold, and (ii) non-ideal sliding motion causes a high-frequency oscillation.

Continuous-time second-order SMCs have been proposed to alleviate the chattering problem [32,33]. However, when continuous-time SMCs are discretized using a sample and held for digital implementation, they may lose their robustness, leading to instability. To address this limitation of continuous-time sliding mode controllers, researchers introduced discrete-time sliding mode controllers (DSMC). Moreover, implementing a DSMC is more straightforward owing to the advancement and widespread availability of computers and microcontrollers. However, the robustness of DSMC is undermined owing to the finite sampling time. In DSMC, the state trajectories converge to a bounded region near the sliding surface called the ultimate band. They exhibit a zigzag motion, termed quasi-sliding mode (QSM). Furthermore, unlike continuous-time SMC, the width of the ultimate band of a DSMC can be determined, which indicates the robustness of the controller. As noted in [34], given the desired ultimate band, the desired controller parameters can be determined; however, the limitations of the controller must be considered. Hence, by manipulating this band, the insensitivity of DSMC to uncertainties can be varied. To increase the robustness and mitigate the chattering effect of conventional DSMC, a second-order DSMC was proposed in [35,36]; however, the controller gains are not optimal.

Although there are a number of research works on DSMC, particularly based on the reaching law [37], its application to USVs has received less attention. This could be because of the difficulty in finding a discretized model of the vehicle [38]. In Ref. [39], a cascaded conventional DSMC and PI-based gain-scheduling controller were used for a straight line following ASV. Different numerical tests were conducted with and without ocean current disturbances. Although the results were satisfactory, the rudder experienced significant chattering.

Therefore, in this study, we propose a discrete-time super-twisting sliding mode controller [35], which is a robust second-order SMC, to address chattering. To the best of our knowledge, DSTA has not been applied to trajectory following for USVs. Additionally, owing to the complexity of obtaining an exact discrete-time model of the vehicle, we assumed certain hydrodynamic parameters and external disturbances, such as ocean currents, waves, and wind, as unknown perturbation. Hence, we applied a time delay estimator (TDE) to estimate the unknown system dynamics.

The TDE estimates the unknown system dynamics based on the past states of the system and control input. It was first introduced by [40] to estimate disturbances in robotic manipulators, and was later applied to underwater control [41,42]. Then, based on this estimation, the controller cancels the uncertainties.

In many previous studies, the effectiveness of the time delay estimator has been experimentally verified in the presence of model uncertainties and external disturbances [36,41,42]. The purpose of the time delay estimator is to assess perturbation by utilizing data from a sufficiently short time ago, aiming to enhance control performance in terms of robustness and control energy. As will be detailed by using equations, the time delay estimator operates under the assumption that the perturbation does not change drastically within one sampling period, even though it dynamically evolves. In our research, the sampling period is 0.01 s, and the derivative of the perturbation remains fairly constant for this duration. This assumption is reflected in the TDE formulation, which can be regarded as the first-order approximation of the Taylor series expansion of the disturbance.

If the time delay estimator is not utilized, higher bounds on uncertainties should be assumed for robust control, leading to an increased control signal. A higher control signal, in turn, indicates higher control energy. Moreover, if the perturbation is not estimated, the controller becomes computationally intensive due to the need to adapt a large number of uncertain parameters. This restricts the real-time use of the control algorithm due to the limited onboard computational resources [43]. These limitations can be addressed using a time delay estimator, which provides information about perturbation to improve the robustness and reduce the control signal [36,41].

**Figure 2:**
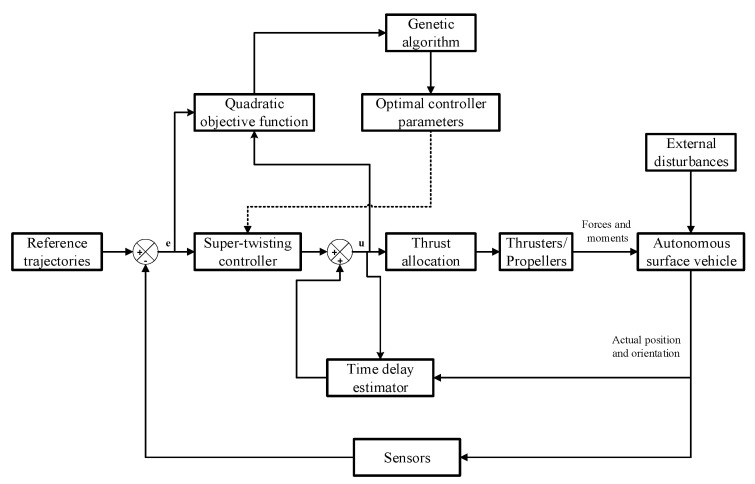
Block diagram of the proposed control method.

Furthermore, the TDE minimizes the time delay, which affects the performance of the sliding mode controller [31]. Finally, we apply a genetic algorithm (GA) to determine the optimal gains of the proposed controller. A quadratic cost function, which is the sum of the tracking error and control energy, was selected. The block diagram of the proposed method is shown in Figure 2. The main contributions of this study are summarized as follows.

1. A robust DSTA has been applied for trajectory following of an ASV. Moreover, a TDE algorithm has been used to estimate the unknown dynamics of the system, which in turn, improves the robustness of the proposed controller.
2. A GA is used to tune the controller parameters based on a quadratic objective function, which is the sum of the tracking error and control energy.
3. A linear matrix inequality (LMI) based Lyapunov approach was employed to validate the stability of the closed-loop system.
4. Finally, simulation results are presented for two scenarios, with and without the presence of exogenous disturbance. Moreover, the performance of the proposed GA based DSTA is compared with that of DSTA and a reaching law based DSMC.

The remainder of this paper is structured as follows. Section 2 describes the mathematical model of the vehicle from which the discretized model is drived using the integral approximation method. Section 3 describes the design of the proposed DSTA controller and the stability of the closed-loop system. Tuning of the parameters of the proposed controller using the GA is presented in Section 4. In Section 5, numerical results and discussions are presented, and finally, Section 6 presents the conclusions.

## 2. Mathematical Model

### 2.1. Continuous-Time Vehicle Model

**Figure 3:**
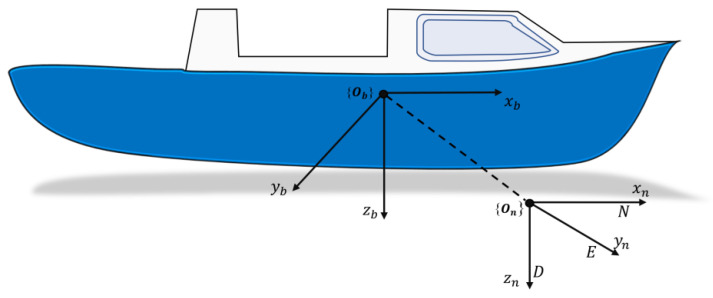
Illustration of body-fixed and earth-fixed reference frames of a surface vehicle.

Generally, the motion of ASVs is described using two coordinate frames of reference [11]: the earth-fixed or inertial frame denoted by $\left\{O_{n}\right\}=\left\{x_{n},y_{n},z_{n}\right\}$, and the body-fixed frame represented by $\left\{O_{b}\right\}=\left\{x_{b},y_{b},z_{b}\right\}$, as shown in Figure 3. Here, only the planar motion of the vehicle was considered. It was assumed that the vehicle had a homogeneous mass distribution and xy-plane symmetry.

The motion of the vehicle in the inertial frame of reference is expressed using position and orientation vector $\eta ={\left[x,y,\psi \right]}^{T}$, whereas its body-fixed velocity is represented by the velocity vector $\nu ={\left[u,v,r\right]}^{T}$. Here, x(m) and y(m) are the positions along $x_{n}$ and $y_{n}$, respectively; ψ(rad) is the yaw angle. Additionally, u(m/s),v(m/s) are the surge and sway velocities along $x_{b}$ and $y_{b}$, respectively, r(rad/s) is the yaw rate about $z_{b}$. Therefore, the kinematic model of the vehicle is given by:

(1) $$\dot{\eta }=J\left(\psi \right)\nu ,$$

where $J\left(\psi \right)\in R^{3\times 3}$ is the transformation matrix between the body-fixed frame and earth-fixed frame, which is:

(2) $$J\left(\psi \right)=\left[\begin{array}{ccc} \cos(\psi ) & -\sin(\psi ) & 0\\ \sin(\psi ) & \cos(\psi ) & 0\\ 0 & 0 & 1 \end{array}\right],$$

and its dynamic model is given by:

(3) $$M\dot{\nu }+C\left(\nu \right)\nu +D\left(\nu \right)\nu =\tau +\tau_{\mathrm{ext}},$$

where $M=\left(M_{\mathrm{RB}}+M_{A}\right)\in R^{3\times 3}$ denotes the sum of the rigid body mass and hydrodynamic added mass; $C\left(\nu \right)=\left(C_{\mathrm{RB}}+C_{A}\right)\in R^{3\times 3}$ is a Coriolis-centripetal matrix due to rigid body and added mass; $D\left(\nu \right)=\left(D_{L}+D_{\mathrm{NL}}\right)\in R^{3\times 3}$ is the sum of linear and nonlinear damping matrices given as follows:

(4a) $$\begin{array}{c} M=\left[\begin{array}{ccc} m_{11} & 0 & 0\\ 0 & m_{22} & m_{23}\\ 0 & m_{32} & m_{33} \end{array}\right], \end{array}$$

(4b) $$\begin{array}{c} C\left(\nu \right)=\left[\begin{array}{ccc} 0 & 0 & c_{13}\\ 0 & 0 & c_{23}\\ c_{31} & c_{32} & 0 \end{array}\right], \end{array}$$

(4c) $$\begin{array}{c} D\left(\nu \right)=\left[\begin{array}{ccc} d_{11} & 0 & 0\\ 0 & d_{22} & d_{23}\\ 0 & d_{32} & d_{33} \end{array}\right], \end{array}$$

where $m_{11}=m-X_{\dot{u}}$, $m_{22}=m-Y_{\dot{v}}$, $m_{23}=mx_{g}-Y_{\dot{r}}$, $m_{32}=mx_{g}-N_{\dot{v}}$, $m_{33}=I_{z}-N_{\dot{r}}$; $c_{13}=-c_{31}=Y_{\dot{v}}v$, $c_{32}=-c_{23}=X_{\dot{u}}$; $d_{11}=-X_{u}-X_{|u|u}$, $d_{22}=-Y_{v}-Y_{|v|v}$, $d_{23}=-Y_{r}-Y_{|r|r}$, $d_{31}=-N_{v}-N_{|v|v}$, $d_{33}=-N_{r}-N_{|r|r}$. Here, *m* is the mass of the vehicle, $I_{z}$ is the moment of inertia about $z_{b}$, and $x_{g}$ is the center of gravity. $X_{\dot{u}}$, $Y_{\dot{v}}$, $Y_{\dot{r}}$, $N_{\dot{v}}$, and $N_{\dot{r}}$ are the hydrodynamic added-mass coefficients. $X_{u}$, $Y_{v}$, $Y_{r}$, $N_{v}$, and $N_{r}$ are constant linear damping coefficients; and, $X_{|u|u}$, $Y_{|v|v}$, $Y_{|r|r}$, $N_{|v|v}$, and $N_{|r|r}$ are constant nonlinear damping coefficients. We assume fore-aft symmetry such that $Y_{\dot{r}}\approx N_{\dot{v}}$. Additionally, for low-speed applications, $N_{v}=Y_{r}$. $\tau ={\left[X,Y,N\right]}^{T}\in R^{3}$ denotes the forces and moments generated by the port thruster, starboard thruster, and bow thruster, respectively. $\tau_{\mathrm{ext}}\in R^{3}$ denote forces and moments due to external disturbances [44]. The response of the thrusters is assumed to be faster than the dynamics of the vehicle; therefore, the dynamics of the thrusters is neglected.

### 2.2. Discrete-Time Vehicle Model

A discrete-time controller can be obtained either by discretizing a continuous-time controller designed using a continuous-time system model or by directly designing a discrete-time controller from the discretized system model. In this study, we followed the latter design approach.

**Assumption** **1.** *Assume that only the rigid body mass ($R_{RB}$) and linear damping ($D_{L}$) of the vehicle are known. The other parameters are unknown, and will be estimated using the TDE. This assumption also helps to decouple the sway and yaw dynamics.*

Thus, based on Assumption 1 the dynamic model of the vehicle given by Equation (1) can be rewritten as follows:

(5) $$M_{\mathrm{RB}}\dot{\nu }\left(t\right)+D_{L}\left(\nu \left(t\right)\right)\nu \left(t\right)+f\left(t\right)=\tau \left(t\right),$$

where $f\left(t\right)=M_{A}\dot{\nu }\left(t\right)+C\left(\nu \left(t\right)\right)\nu \left(t\right)+D_{\mathrm{NL}}\left(\nu \left(t\right)\right)\nu \left(t\right)-\tau_{\mathrm{ext}}\left(t\right)$ is unknown or perturbation term to be estimated.

**Assumption** **2.** *The state variables and control input are assumed to be constant during the sampling time period T, that is, v(t) and τ(t) remain constant* [45].

Using Assumption 2, we compute a discrete-time model of the vehicle. By integrating Equation (5) over the sampling interval kT into (k+1)T, we obtain:

$$\begin{array}{c} \int_{kT}^{(k+1)T}M_{\mathrm{RB}}\dot{\nu }\left(t\right)\;dt+\int_{kT}^{(k+1)T}D_{L}\left(\nu \left(t\right)\right)\nu \left(t\right)\;dt+\int_{kT}^{(k+1)T}f\left(t\right)\;dt=\int_{kT}^{(k+1)T}\tau \left(t\right)\;dt, \end{array}$$

(6) $$\begin{array}{c} M_{\mathrm{RB}}(\nu \left(\left(k+1\right)T\right)-\nu \left(kT\right)))+TD_{L}\nu \left(kT\right)+\int_{kT}^{(k+1)T}f\left(t\right)\;dt=T\tau \left(kT\right). \end{array}$$

In Equation (6), f(t) contains the terms that cannot be integrated. After rearranging Equation (6), the following discretized expression is obtained:

(7) ν(k+1)=Aν(k)+Bτ(k)+Γ(k),

where $\nu \left(k\right)={\left[u\left(k\right),v\left(k\right),r\left(k\right)\right]}^{T}$; $\tau \left(k\right)={\left[X_{k},Y_{k},N_{k}\right]}^{T}$; the system matrix $A=I-TM_{\mathrm{RB}}D_{L}$; an input matrix $B=TM_{\mathrm{RB}}^{-1}$; $\Gamma \left(k\right)=M_{\mathrm{RB}}^{-1}\int_{kT}^{(k+1)T}f\left(t\right)\;dt$. Γ(k) denotes, as explained in *Assumption 1*, hydrodynamics and parameter uncertainties and the external disturbance. I denotes the identity matrix of appropriate dimension.

**Assumption** **3.** *The perturbation term $\Gamma \left(k\right)\in R^{3}$ satisfies the Lipschitz continuity condition*

$$∥\Gamma \left(k\right)-\Gamma \left(k-1\right)∥\leq TL_{\gamma },$$

*where $L_{\gamma }>0$ is a Lipschitz constant and T is the sampling time* [46].

## 3. Controller Design and Stability Analysis

In this section, the proposed DSTA controller design and stability analysis using the Lyapunov approach are discussed.

### 3.1. Controller Design

The control problem involves designing a robust controller such that the vehicle follows a reference or desired trajectory while addressing the tracking error and chattering effect. In this study, the desired trajectories were defined in a body-fixed reference frame, that is, a body-fixed velocity trajectory following.

To design a SMC, the first step is to select the sliding surface to which the system trajectories are forced to converge. Hence, let the desired trajectories be $\nu_{d}\left(k\right)={\left[u_{d}\left(k\right),v_{d}\left(k\right),r_{d}\left(k\right)\right]}^{T}$. The desired trajectories are generated by the guidance system. Also, let the actual trajectories be $\nu \left(k\right)={\left[u\left(k\right),v\left(k\right),r\left(k\right)\right]}^{T}$ provided by the navigation system. Thus, the trajectory error is the difference between the desired and the actual trajectories, that is, $e\left(k\right)=\nu_{d}\left(k\right)-\nu \left(k\right)$. The sliding surface was selected as follows:

(8) $$s\left(k\right)=K_{p}e\left(k\right),$$

where $s\left(k\right)\in R^{3}$; $K_{p}\in R^{3\times 3}$ is positive scalar diagonal matrix.

For the trajectories to converge to the sliding surface, the sliding function must satisfy s(k+1)=s(k)=0 after some time instant *k*; however, this does not occur in the real world, instead, the system trajectories are confined to a band near the sliding manifold, that is, QSM. For convergence of the system trajectories, the sliding function must satisfy ∥s(k+1)∥ < ∥s(k)∥. We compute s(k+1) from Equation (8) as follows:

(9) $$\begin{array}{cc} s(k+1) & =K_{p}e\left(k+1\right)\\ \; & =K_{p}\left\{\nu_{d}\left(k+1\right)-\nu \left(k+1\right)\right\} \end{array}$$

Now, by substituting Equation (7) into Equation (9), we obtain

(10) $$\begin{array}{cc} s(k+1) & =K_{p}\left\{\nu_{d}\left(k+1\right)-A\nu \left(k\right)-B\tau \left(k\right)-\Gamma \left(k\right)\right\}. \end{array}$$

The DSTA is given by [35]

(11) $$\begin{array}{cc} s(k+1) & =K_{1}s\left(k\right)-T\Omega_{1}{\left|s\left(k\right)\right|}^{1/2}\mathrm{sgn}\left(s\left(k\right)\right)+T\varsigma \left(k\right)\\ \varsigma (k+1) & =K_{2}\varsigma \left(k\right)-T\Omega_{2}\mathrm{sgn}\left(s\left(k\right)\right), \end{array}$$

where $K_{i}\in R^{3\times 3}$, $0<(\mathrm{diagonal}\;\mathrm{elements}\;\mathrm{of}\;K_{i})<1$, and $\Omega_{i}\in R^{3\times 3}$ for i=1,2 are diagonal matrices to be designed to ensure the convergence of DSTA. *T* is the sampling time period, and sgn(s(k)) is a signum function defined as

$$\mathrm{sgn}\left(\gamma \right)=\left\{\begin{array}{ccc} 0, & \mathrm{for} & \gamma =0\\ \frac{\gamma }{\left|\gamma \right|}, & \mathrm{for} & \gamma \neq 0. \end{array}\right.$$

where γ∈R. By equating Equation (10) with Equation (11), the control law becomes:

(12) $$\begin{array}{cc} \tau (k) & ={\left(K_{p}B\right)}^{-1}\{K_{p}\nu_{d}\left(k+1\right)-K_{p}A\nu \left(k\right)-K_{p}\Gamma \left(k\right)+\\ \; & -K_{1}s\left(k\right)+T\Omega_{1}{\left|s\left(k\right)\right|}^{1/2}\mathrm{sgn}\left(s\left(k\right)\right)-T\varsigma \left(k\right)\}, \end{array}$$

where ${\left(K_{p}B\right)}^{-1}$ must exist. The control law given by Equation (12) cannot be realized, because the perturbation term Γ(k) is unknown. From Assumption 3, the perturbation term is bounded. Hence, we employ the TDE method to estimate the perturbation term such that $\hat{\Gamma }\left(k\right)$ is used instead of Γ(k). The control law can be rewritten as follows:

(13) $$\begin{array}{cc} \tau (k) & ={\left(K_{p}B\right)}^{-1}\{K_{p}\nu_{d}\left(k+1\right)-K_{p}A\nu \left(k\right)-K_{p}\hat{\Gamma }\left(k\right)-K_{1}s\left(k\right)\\ \; & +T\Omega_{1}{\left|s\left(k\right)\right|}^{1/2}\mathrm{sgn}\left(s\left(k\right)\right)-T\varsigma \left(k\right)\}. \end{array}$$

The perturbation term is estimated based on the values of the past state variables of the system and past control inputs, that is, the past dynamics of the system. Mathematically, this can be expressed as $\Gamma \left(k\right)=\hat{\Gamma }\left(k\right)=\Gamma \left(k-1\right)$. The estimation of the perturbation term $\hat{\Gamma }\left(k\right)$ can be obtained from Equation (7) as follows:

(14) $$\hat{\Gamma }\left(k\right)=\Gamma \left(k-1\right)=\nu \left(k\right)-A\nu \left(k-1\right)-B\tau \left(k-1\right).$$

The estimation in Equation (14) is based on the assumption that the perturbation term does not vary significantly during the sampling time. However, if the perturbation term changes rapidly during sampling time, the estimation error may be large [45]. Hence, the estimation can be further improved if the delayed perturbation term Γ(k−1) and differential of Γ(k−1), which is ΔΓ(k−1), are used to estimate the perturbation term. Now, we get:

(15) $$\begin{array}{cc} \hat{\Gamma }\left(k\right) & =\Gamma (k-1)+\Delta \Gamma (k-1)\\ \; & =2\Gamma (k-1)-\Gamma (k-2). \end{array}$$

After substituting Equation (14) into Equation (15), the estimated perturbation term becomes

(16) $$\begin{array}{cc} \hat{\Gamma }\left(k\right) & =2\nu (k)-\nu (k-1)-2A\nu (k-1)+A\nu (k-2)\\ \; & -2B\tau (k-1)+B\tau (k-2). \end{array}$$

Therefore, by substituting the perturbation term Equation (16) into Equation (13), we obtain the following control law:

(17) $$\begin{array}{cc} \tau (k) & ={\left(K_{p}B\right)}^{-1}\{K_{p}\nu_{d}\left(k+1\right)-K_{p}A\nu \left(k\right)-2K_{p}\nu \left(k\right)+K_{p}\nu \left(k-1\right)+2K_{p}A\nu \left(k-1\right)\\ \; & -K_{p}A\nu \left(k-2\right)-K_{1}s\left(k\right)+T\Omega_{1}{\left|s\left(k\right)\right|}^{1/2}\mathrm{sgn}\left(s\left(k\right)\right)-T\varsigma \left(k\right)\}+2\tau \left(k-1\right)\\ \; & -\tau (k-2). \end{array}$$

The control law given by Equation (17) can be implemented easily because the perturbation term is known. The delayed state and control input of the system can be retrieved from computer memory.

### 3.2. Stability Analysis

The stability of a closed-loop system is provided as follows [35,36,47]:

**Theorem** **1.** *Consider the dynamics of the surface vehicle given by Equation (2). If there exists a positive definite solution $L=L^{T}>0$ to an LMI $\Psi^{T}\left[L+L\Lambda_{1}L+L\Lambda_{2}L\right]\Psi -\left(1-r\right)L\leq -\Psi$, the conditions $\Omega_{1i}<0$, and $\Omega_{2i}>0$ are met. Therefore, the proposed DSTA controller given by Equation (17) ensures asymptotic convergence of the system trajectories to a ball $B_{r}$ (that is, $B_{r}=\left\{s:{∥s∥}^{2}<r_{b}\right\}$) centered at the origin. The ball has a radius of convergence.*

$$r_{b}=\frac{\sigma }{1-r},$$

*where 0<r<1 is the ball radius, s is the sliding surface, and*

$$\sigma =\frac{{\bar{\varrho_{1}}}^{2}}{4\zeta_{1}}+\zeta_{2}\epsilon_{i}^{2}+\bar{\varrho_{2}},$$

$${\bar{\varrho }}_{1}=\varrho_{1}+\varrho_{2}=T^{2}\Omega_{1i}^{2}f_{11}+2T^{3}f_{12}\Omega_{1i}\Omega_{2i},$$

$${\bar{\varrho }}_{2}=\varrho_{2}+\varrho_{3}=T^{2}\Omega_{1i}^{2}f_{11}+T^{4}f_{22}\Omega_{2i},$$

$$\zeta_{1}=\lambda_{\min}\left(\Psi \right),$$

$$\zeta_{2}=\lambda_{\max}\left(H\right).$$

**Proof of Theorem** **1.** The closed-loop system dynamics can be obtained by substituting Equation (17) into Equation (10), and we obtain

(18) $$\begin{array}{ll} s(k+1) & =K_{1}s\left(k\right)-T\Omega_{1}{\left|s\left(k\right)\right|}^{1/2}\mathrm{sgn}\left(s\left(k\right)\right)+T\varsigma \left(k\right)+\Delta \Gamma \left(k\right)\\ \varsigma (k+1) & =K_{2}\varsigma \left(k\right)-T\Omega_{2}\mathrm{sgn}\left(s\left(k\right)\right), \end{array}$$

where $\Delta \Gamma \left(k\right)=\hat{\Gamma }\left(k\right)-\Gamma \left(k\right)$ denotes the estimation error. As shown in Equation (18), the estimation error affects the closed-loop dynamics of the system. In the following section, we verify the robustness of the proposed controller by assuming that the perturbation term satisfies the Lipschitz criterion, as explained in Assumption 3. Now, we rearrange Equation (18) using change of variable. Let ρ(k)=Tς(k)+ΔΓ(k). From this, we obtain

(19) $$\begin{array}{ll} s(k+1) & =K_{1}s\left(k\right)-T\Omega_{1}{\left|s\left(k\right)\right|}^{1/2}\mathrm{sgn}\left(s\left(k\right)\right)+\rho \left(k\right)\\ \rho (k+1) & =K_{2}\rho \left(k\right)-T^{2}\Omega_{2}\mathrm{sgn}\left(s\left(k\right)\right)+\epsilon \left(k\right), \end{array}$$

where $\epsilon \left(k\right)=\Delta \Gamma \left(k+1\right)-K_{2}\Delta \Gamma \left(k\right)$. Without loss of generality, we can rewrite Equation (19) for i=1,2,3 as:

(20) $$\begin{array}{ll} s_{i}\left(k+1\right) & =K_{1i}s_{i}\left(k\right)-T\Omega_{1i}{\left|s_{i}\left(k\right)\right|}^{1/2}\mathrm{sgn}\left(s_{i}\left(k\right)\right)+\rho_{i}\left(k\right)\\ \rho_{i}\left(k+1\right) & =K_{2i}\rho_{i}-T^{2}\Omega_{2i}\mathrm{sgn}\left(s_{i}\left(k\right)\right)+\epsilon_{i}\left(k\right). \end{array}$$

Equation (20) can be expressed using a compact matrix notation as follows:

(21) $$\chi_{i}\left(k+1\right)=\Psi \left(k\right)\chi_{i}\left(k\right)+\varphi \left(k\right)\mathrm{sgn}\left(s_{i}\left(k\right)\right)+\xi \left(k\right),$$

where $\chi_{i}\left(k\right)={\left[s_{i}\left(k\right),\rho_{i}\left(k\right)\right]}^{T}$, and Ψ(k), φ(k), and ξ(k) are:

(22a) $$\begin{array}{r} \Psi \left(k\right)=\left[\begin{array}{cc} K_{1i} & 1\\ 0 & K_{2i} \end{array}\right], \end{array}$$

(22b) $$\begin{array}{l} \varphi \left(k\right)=\left[\begin{array}{c} -T\Omega_{1i}\mathrm{sgn}{\left|s_{i}\left(k\right)\right|}^{1/2}\\ -T^{2}\Omega_{2i} \end{array}\right], \end{array}$$

(22c) $$\begin{array}{r} \xi \left(k\right)=\left[\begin{array}{c} 0\\ \epsilon_{i}\left(k\right) \end{array}\right]. \end{array}$$

We select a positive definite Lyapunov function V(k) that satisfies $\lambda_{\min}\left(L\right)∥\chi_{i}{\left(k\right)∥}^{2}\;\leq \;∥\chi_{i}{\left(k\right)∥}_{L}\leq \lambda_{\max}\left(L\right){∥\chi_{i}\left(k\right)∥}^{2}$. Here, $\lambda_{\min}$ and $\lambda_{\max}$ are the minimum and maximum eigenvalues of L, respectively.

(23) $$V_{i}\left(k\right)=\chi_{i}^{T}\left(k\right)L\chi_{i}\left(k\right).$$

The difference in the Lyapunov function is:

(24) $$\Delta V_{i}=V_{i}\left(k+1\right)-V_{i}\left(k\right).$$

By substituting Equation (21) into Equation (24), we obtain

(25) $$\begin{array}{ll} \Delta V_{i} & =V_{i}\left(k+1\right)-V_{i}\left(k\right)\\ & =\chi_{i}^{T}\left(k+1\right)L\chi_{i}\left(k+1\right)-\chi_{i}^{T}\left(k\right)L\chi_{i}\left(k\right), \end{array}$$

Substituting Equation (24) into Equation (25), we obtain

(26) $$\begin{array}{ll} \Delta V_{i}\left(k\right) & =\chi_{i}^{T}\left(k\right)\Psi^{T}L\Psi \chi_{i}\left(k\right)+\chi_{i}^{T}\left(k\right)\Psi^{T}L\varphi \mathrm{sgn}\left(s_{i}\left(k\right)\right)+\chi_{i}^{T}\left(k\right)\Psi^{T}L\xi +\varphi^{T}L\Psi \chi_{i}\left(k\right)\mathrm{sgn}\left(s_{i}\left(k\right)\right)\\ & +\varphi^{T}L\varphi +\varphi^{T}L\xi \mathrm{sgn}\left(s_{i}\left(k\right)\right)+\xi^{T}L\Psi \chi_{i}\left(k\right)+\xi^{T}L\varphi \mathrm{sgn}\left(s_{i}\left(k\right)\right)+\xi^{T}L\xi -\chi_{i}^{T}\left(k\right)L\chi_{i}\left(k\right). \end{array}$$

After collecting these terms, Equation (26) becomes

(27) $$\begin{array}{ll} \Delta V_{i}\left(k\right) & =\chi_{i}^{T}\left(k\right)\left\{\Psi^{T}L\Psi -L\right\}\chi_{i}\left(k\right)+\varphi^{T}L\varphi +\xi^{T}L\xi +2\chi_{i}^{T}\left(k\right)\Psi^{T}L\varphi \left(k\right)\mathrm{sgn}\left(s_{i}\left(k\right)\right)\\ & +2\chi_{i}^{T}\left(k\right)\Psi^{T}L\xi +2\varphi^{T}L\xi \mathrm{sgn}\left(s_{i}\left(k\right)\right). \end{array}$$

Using the Λ-matrix inequality ${\bar{A}}^{T}\bar{B}+{\bar{B}}^{T}\bar{A}\leq {\bar{A}}^{T}\Lambda \bar{A}+{\bar{B}}^{T}\Lambda^{-1}\bar{B}$ [48], some of the expressions in Equation (27) are simplified as follows:

1. $\;\begin{array}{ll} 2\chi_{i}^{T}\left(k\right)\Psi^{T}L\varphi \mathrm{sgn}\left(s_{i}\left(k\right)\right) & \leq \chi_{i}^{T}\left(k\right)\left\{\Psi^{T}L\Lambda_{1}L\Psi \right\}\chi_{i}\left(k\right)+\varphi^{T}\Lambda_{1}^{-1}\varphi . \end{array}$
2. $\;\begin{array}{l} \chi_{i}^{T}\left(k\right)\Psi^{T}L\xi \leq \chi_{i}^{T}(k\left\{\Psi^{T}L\Lambda_{2}L\Psi \right\}\chi_{i}\left(k\right)+\xi^{T}\Lambda_{2}^{-1}\xi . \end{array}$
3. $\;\begin{array}{l} \xi^{T}L\xi \mathrm{sgn}\left(s_{i}\left(k\right)\right)\leq \varphi^{T}L\Lambda_{3}L\varphi +\xi^{T}\Lambda_{3}^{-1}\xi . \end{array}$

Hence, Equation (27) can be rewritten as

(28) $$\begin{array}{ll} \Delta V_{i}\left(k\right) & \leq \chi_{i}^{T}\left(k\right)\left\{\Psi^{T}L\Psi +\Psi^{T}L\Lambda_{1}L\Psi +\Psi^{T}L\Lambda_{2}L\Psi -L\right\}\chi_{i}\left(k\right)+\varphi^{T}\left\{L+\Lambda_{1}^{-1}+L\Lambda_{3}L\right\}\varphi \\ \; & +\xi^{T}\{L+\Lambda_{2}^{-1}+\Lambda_{3}^{-1}\}\xi . \end{array}$$

By adding $rV_{i}\left(k\right)$ to both sides of Equation (28), we obtain

(29) $$\begin{array}{ll} \Delta V_{i}\left(k\right) & \leq \chi_{i}^{T}\left(k\right)\left\{\Psi^{T}\left\{L+L\Lambda_{1}L+L\Lambda_{2}L-\left(1-r\right)L\right\}\Psi \right\}\chi_{i}\left(k\right)+\varphi^{T}\{L+\Lambda_{1}^{-1}\\ \; & +L\Lambda_{3}L]\varphi +\xi^{T}\left\{L+\Lambda_{2}^{-1}+\Lambda_{3}^{-1}\right\}\xi -rV_{i}\left(k\right), \end{array}$$

where 0<r<1. Using LMI, if there exists a negative definite matrix Φ satisfying the following inequality:

$$\Psi^{T}\left\{L+L\Lambda_{1}L+L\Lambda_{2}L\left(1-r\right)L\right\}\Psi \leq -\Phi ,$$

$\Delta V_{i}\left(k+1\right)$ can be rewritten as

(30) $$\begin{array}{ll} \Delta V_{i}\left(k\right) & \leq \chi_{i}^{T}\left(k\right)\Phi \chi_{i}\left(k\right)+\varphi^{T}\left\{L+\Lambda_{1}^{-1}+L\Lambda_{3}L\right\}\varphi +\xi^{T}\left\{L+\Lambda_{2}^{-1}+\Lambda_{3}^{-1}\right\}\xi -rV_{i}\left(k\right). \end{array}$$

(31) $$\begin{array}{l} \Delta V_{i}\left(k\right)\leq -\chi_{i}^{T}\left(k\right)\Phi \chi_{i}\left(k\right)+\varphi^{T}F\varphi +\xi^{T}H\xi -rV_{i}\left(k\right), \end{array}$$

where $F=L+\Lambda_{1}^{-1}+L\Lambda_{3}L$, and $H=L+\Lambda_{2}^{-1}+\Lambda_{3}^{-1}$.

(32) $$\begin{array}{l} \Delta V_{i}\left(k\right)\leq -\chi_{i}^{T}\left(k\right)\Phi \chi_{i}\left(k\right)+\varphi^{T}F\varphi +\xi^{T}H\xi -rV_{i}\left(k\right), \end{array}$$

However, $\varphi^{T}F\varphi$ can be simplified as follows [36]:

(33) $$\varphi^{T}F\varphi =\bar{\varrho_{1}}\left|s_{i}\left(k\right)\right|+\bar{\varrho_{2}}.$$

The simplification of Equation (33) is presented in Appendix A. For the positive definite matrices Ψ and H in Equation (31), we can apply the two-norm definition: $\lambda_{\min}{\left(\Psi \right)∥\chi \left(k\right)∥}^{2}\leq ∥\chi_{i}{\left(k\right)∥}_{\Psi }\leq \lambda_{\max}\left(\Psi \right){∥\chi_{i}\left(k\right)∥}^{2}$, and $\lambda_{\min}{\left(H\right)∥\xi \left(k\right)∥}^{2}\leq {∥\xi \left(k\right)∥}_{H}\leq \lambda_{\max}\left(H\right){∥\xi \left(k\right)∥}^{2}$. Here, $\lambda_{\min}$ and $\lambda_{\max}$ are the minimum and maximum eigenvalues of the matrices, respective. Thus, Equation (32) becomes

(34) $$\begin{array}{ll} \Delta V_{i}\left(k\right) & \leq -\chi_{i}^{T}\left(k\right)\Phi \chi_{i}\left(k\right)+\varphi^{T}F\varphi +\xi^{T}H\xi -rV_{i}\left(k\right)\leq -\lambda_{\min}\left(\Psi \right)∥\chi_{i}{\left(k\right)∥}^{2}+\bar{\varrho_{1}}\left|s_{i}\left(k\right)\right|+\bar{\varrho_{2}}\\ \; & +\lambda_{\max}{\left(H\right)∥\xi \left(k\right)∥}^{2}-rV_{i}\left(k\right)\leq -\lambda_{\min}\left(\Psi \right)∥\chi_{i}{\left(k\right)∥}^{2}+\bar{\varrho_{1}}\left|s_{i}\left(k\right)\right|+\bar{\varrho_{2}}\\ \; & +\lambda_{\max}\left(H\right)\epsilon_{i}^{2}-rV_{i}\left(k\right). \end{array}$$

Let $\zeta_{1}=\lambda_{\min}\left(\Psi \right)>0$, and $\zeta_{2}=\lambda_{\max}\left(H\right)$. $|s_{i}\left(k\right)|\leq ∥\chi_{i}\left(k\right)∥$, Equation (34) becomes

(35) $$\begin{array}{ll} \Delta V_{i}\left(k\right) & \leq -\zeta_{1}∥\chi_{i}{\left(k\right)∥}^{2}+\bar{\varrho_{1}}\left|s_{i}\left(k\right)\right|+\bar{\varrho_{2}}\\ \; & +\zeta_{2}\epsilon_{i}^{2}-rV_{i}\left(k\right)\\ \; & \leq -\zeta_{1}∥\chi_{i}{\left(k\right)∥}^{2}+\bar{\varrho_{1}}∥\chi_{i}\left(k\right)∥+\zeta_{2}\epsilon_{i}^{2}\\ \; & +\bar{\varrho_{2}}-rV_{i}\left(k\right). \end{array}$$

The expression $\zeta_{1}∥\chi_{i}{\left(k\right)∥}^{2}+\bar{\varrho_{1}}∥\chi_{i}\left(k\right)∥$ in Equation (35) is simplified using the completing squares method as follows:

(36) $$\begin{array}{ll} -\zeta_{1}∥\chi_{i}{\left(k\right)∥}^{2}+\bar{\varrho_{1}}∥\chi_{i}\left(k\right)∥ & =-\zeta_{1}\left\{∥\chi_{i}{\left(k\right)∥}^{2}-\frac{\bar{\varrho_{1}}}{\zeta_{1}}∥\chi_{i}\left(k\right)∥\right\}\\ \; & =-\zeta_{1}{\left\{∥\chi_{i}\left(k\right)∥-\frac{\bar{\varrho_{1}}}{2\zeta_{1}}\right\}}^{2}+\frac{{\bar{\varrho_{1}}}^{2}}{4\zeta_{1}}. \end{array}$$

By substituting Equation (36) into Equation (35), we obtain

(37) $$\begin{array}{ll} \Delta V_{i}\left(k\right) & \leq -\zeta_{1}∥\chi_{i}{\left(k\right)∥}^{2}+\bar{\varrho_{1}}∥\chi_{i}\left(k\right)∥+\zeta_{2}\epsilon_{i}^{2}+\bar{\varrho_{2}}-rV_{i}\left(k\right)\\ \; & \leq -\zeta_{1}{\left\{∥\chi_{i}\left(k\right)∥-\frac{\bar{\varrho_{1}}}{2\zeta_{1}}\right\}}^{2}+\frac{{\bar{\varrho_{1}}}^{2}}{4\zeta_{1}}+\zeta_{2}\epsilon_{i}^{2}+\bar{\varrho_{2}}-rV_{i}\left(k\right)\left(k\right)\\ \; & \leq \frac{{\bar{\varrho_{1}}}^{2}}{4\zeta_{1}}+\zeta_{2}\epsilon_{i}^{2}+\bar{\varrho_{2}}-rV_{i}\\ \; & =\sigma -rV_{i}\left(k\right), \end{array}$$

where $\sigma =\frac{{\bar{\varrho_{1}}}^{2}}{4\zeta_{1}}+\zeta_{2}\epsilon_{i}^{2}+\bar{\varrho_{2}}$.

(38) $$\begin{array}{ll} \Delta V_{i}\left(k\right) & \leq \sigma -rV_{i}\\ V_{i}\left(k+1\right)-V_{i}\left(k\right) & \leq \sigma -rV_{i}\left(k\right)\\ V_{i}\left(k+1\right) & \leq \left(1-r\right)V_{i}\left(k\right)+\sigma . \end{array}$$

Equation (38) is a linear, first-order, ordinary difference equation; thus, we can compute the solution by induction from the first few iterations given as follows:

(39a) $$\begin{array}{ll} V_{i}\left(1\right) & \leq \sigma +\left(1-r\right)V_{i}\left(0\right), \end{array}$$

(39b) $$\begin{array}{ll} V_{i}\left(2\right) & \leq \sigma +\sigma \left(1-r\right)+{\left(1-r\right)}^{2}V_{i}\left(0\right), \end{array}$$

(39c) $$\begin{array}{ll} V_{i}\left(3\right) & \leq \sigma +\sigma \left(1-r\right)+\sigma {\left(1-r\right)}^{2}+\sigma {\left(1-r\right)}^{3}V_{i}\left(0\right). \end{array}$$

Therefore, from the results in Equations (39a)–(39c), we can generalize the kth iteration as follows:

(40) $$\begin{array}{ll} V_{i}\left(k\right) & \leq \sigma +\sigma \left(1-r\right)V_{i}\left(0\right)\\ \; & \leq \sigma +\sigma \left(1-r\right)+\sigma {\left(1-r\right)}^{2}+\cdots +\sigma {\left(1-r\right)}^{k-1}+{\left(1-r\right)}^{k}V_{i}\left(0\right)\\ \; & \leq {\left(1-r\right)}^{k}V_{i}\left(0\right)+\sum_{j=0}^{k-1}\sigma {\left(1-r\right)}^{j}. \end{array}$$

As *k* approaches infinity, $V_{i}\left(k\right)$ becomes:

(41) $$\lim_{k\to \infty }V_{i}\left(k\right)\leq \frac{\sigma }{1-r}.$$

Hence, the radius of the ball is

$$r_{b}\leq \frac{\sigma }{1-r}.$$

This concludes the proof of Theorem 1. □

## 4. Controller Gain Optimization Using Genetic Algorithm

In this section, the computation of the optimal gains of the proposed controller using GA is discussed. Setting controller gains, particularly for complex nonlinear systems, is difficult and requires experience. The system response can be made faster with higher controller gains; however, this leads to higher control signals, which in turn increase the energy consumption and actuator saturation. Thus, to address these problems, we applied a GA to tune the controller gains.

To determine optimal gains, an objective or cost function must first be established. In optimal control, the choice of cost function depends on the performance measures that the system must satisfy. These performance measures can be the error, time, and control energy, or a combination of them [49]. The use of the sum of tracking error and control energy, or a combination of other performance metrics as a cost function, is a common approach and has been applied in real-world applications. Nevertheless, there is always a trade-off between the selections of the performance criteria. After selecting the cost function, a suitable optimization algorithm is chosen to find the optimal parameters of the controller based on the cost function.

**Figure 4:**
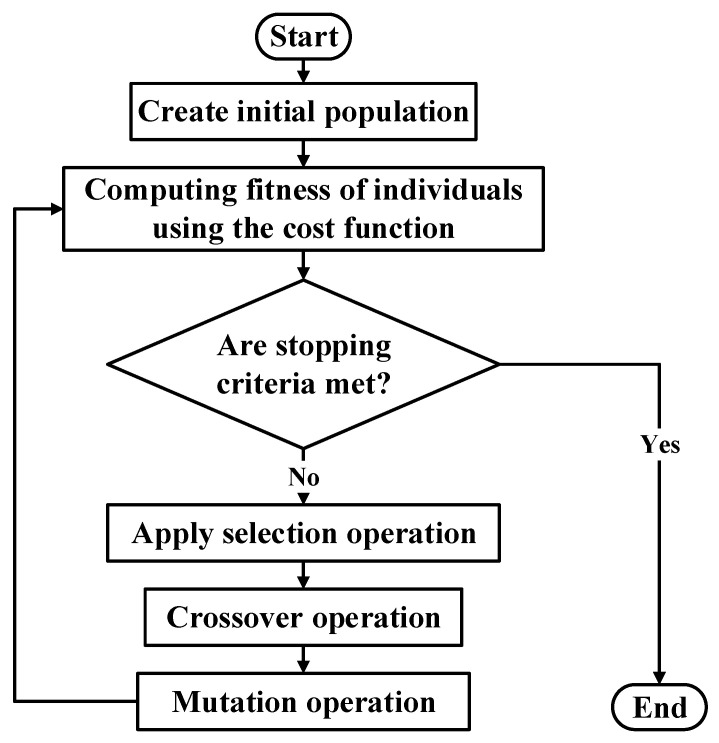
Flowchart of the Genetic algorithm for gain optimization.

In this study, GA was selected because it is convenient for discrete-time optimization [50]. GA is an evolutionary algorithm based on Darwin’s theory of natural selection. Generally, it uses three operators: selection, crossover, and mutation. The operation of the GA commences by setting an initial random population comprising possible candidate solutions, and the fitness of the individuals is evaluated using the cost function. The selection operator then selects the fittest individuals, from which parents are selected for reproduction. Offsprings are produced by crossing the chromosomes of the parents. To increase the diversity of the next generation, a mutation operator is applied that prevents stagnation, that is, the similarity of the solution after several generations. This operation continues until the stopping criteria are satisfied [50,51]. The GA operation is summarized in the flowchart in Figure 4.

The proposed method avoids real-time use of GA. If employed in real-time, the convergence time of GA significantly impacts control performance. Problems may arise if GA fails to converge within the desired time period corresponding to the control frequency. To address this, we employed GA to tune the controller parameters offline before real-time operation. This tuning is specifically for the nominal case, without external disturbances, and is done prior to the real-time operation of the controller.

Since ASVs can stay on the water surface for long periods of time, they should use energy efficiently apart from following the desired trajectory by minimizing the tracking error. Thus, we employ a quadratic cost function $J_{c}$, which is the sum of the tracking error and control energy, defined as follows:

(42) $$J_{c}=\sum_{i=0}^{k-1}{(∥e\left(i\right)∥}_{Q}^{2}+{∥u\left(i\right)∥}_{R}^{2}),$$

where *k* is the number of time steps and e(i) and u(i) are the vectors of the tracking error and control signal at the $i^{\mathrm{th}}$ time instant, respectively. $Q\in R^{3\times 3}$ is a positive semi-definite matrix and $R\in R^{3\times 3}$ is a positive definite matrix. The cost function, as defined in Equation (42), incorporates weighting matrices Q and R for the tracking error index and control energy index, respectively. The values of these matrices depend on the size of the tracking error and the control energy, as mentioned in [52]. They are selected to strike a balance between tracking performance and control energy consumption. It should be noted the cost function is formulated as an unconstrained optimization problem.

The cost function given by Equation (42) is coded using MATLAB. The error and control signals are logged into the MATLAB workspace using the Simulink^®^ model implementation of the vehicle. The cost function is computed from these signals. Then, the cost function and GA options were passed to the built-in MATLAB GA. Finally, the algorithm tunes the controller’s gains until the stopping criteria are reached, which in our case are the maximum number of generations and stall generations.

**Figure 5:**
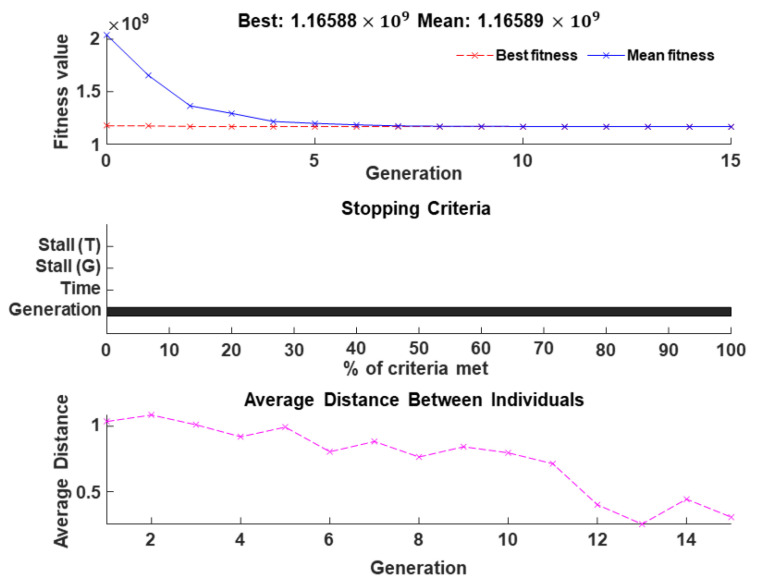
Illustration of convergence of genetic algorithm using fitness, stopping criteria, and average distance between individuals.

Figure 5 illustrates our analysis of GA convergence, showing plots of fitness, stopping criteria, and the average distance between individuals. The GA operates with a population size of 250, and its stopping criteria include a maximum of 15 generations and a stall generation of 7, as outlined in Table 1. Other GA settings are detailed in the same table. The fitness graph in Figure 5 demonstrates GA convergence to the ‘best’ solution in nearly 6 generations, terminating based on the maximum number of generations criterion. Moreover, the graph depicting the average distance between individuals gradually decreases as the generation advances, indicating the convergence of GA towards a proper solution.

## 5. Simulation Results and Discussion

This section presents a verification of the performance of the proposed controller through numerical simulations. The simulation comprises two scenarios: (i) the performances of the proposed GA-based DSTA (hereafter, GA-DSTA) and DSTA controllers with TDE are compared. Additionally, to evaluate the performance of these controllers and chattering reduction, we used the commonly used conventional discrete-time SMC based on reaching law (DSMR) implemented in [45]. (ii) Since ASVs could be deployed in areas where the disturbance due to ocean current, wind, and ocean waves is significant, the robustness of the proposed controller will be tested in the presence of an external disturbance.

The reference trajectories used were body-fixed velocity trajectories. The physical parameters of the vehicle are obtained from the research vessel used in [44]. The vehicle has an overall length of 2.53 m and a 0.565 m beam, and is a scaled-down (1:17.1) model of a large research vessel. The vehicle parameters are listed in Table 2.

### 5.1. Scenario 1: Nominal Case

In this case, the performance of the proposed controller without external disturbances is addressed. A piecewise body-fixed reference trajectory given in Equations (43)–(45) for surge, sway, and yaw was used to test the effectiveness of the proposed controller. Discontinuous trajectories are used to validate the controller’s performance in worst-case scenarios, even though they may not be used in real-world scenarios. To assess the dynamic performance of the controller, i.e., how the controller reacts to abrupt changes in trajectory, abrupt changes have been considered. These trajectories were discretized using the sample-and-hold method. We assume that the maximum surge speed, sway speed, and yaw rate of the vehicle are 15 m/s, 2 m/s, and 1 rad/s, respectively. The initial speed of the vehicle is set to ${\left[u_{0},v_{0},r_{0}\right]}^{T}$ = ${\left[0\;m/s,0\;m/s,0\;\mathrm{rad}/s\right]}^{T}$. A sampling time has to be selected based on the update rate or frequency of the sensors and actuators. Thus, in this study, a sampling time of *T* = 0.01 s is chosen.

(43) $$u_{d}=\left\{\begin{array}{ccc} 0.1t^{2}, & \mathrm{for} & 0\leq t<10\\ -10\sin(0.1t)+5\cos(0.1\pi t), & \mathrm{for} & 10\leq t<20\\ 0.01t^{2}-2\sin\left(0.05\pi t\right), & \mathrm{for} & 20\leq t<30\\ 100e^{-\frac{t}{8}}\sin\left(0.8t\right), & \mathrm{for} & 30\leq t<40 \end{array}\right.$$

(44) $$v_{d}=\left\{\begin{array}{ccc} 0, & \mathrm{for} & 0\leq t<10\\ 2\sin(0.05\pi t), & \mathrm{for} & 10\leq t<20\\ -1.5, & \mathrm{for} & 20\leq t<30\\ 0.8\sin(0.5t), & \mathrm{for} & 30\leq t<40 \end{array}\right.$$

(45) $$r_{d}=\left\{\begin{array}{ccc} \frac{\pi }{4}, & \mathrm{for} & 0\leq t<10\\ 0.4, & \mathrm{for} & 10\leq t<20\\ e^{-\frac{t}{8}}\sin\left(0.8t\right), & \mathrm{for} & 20\leq t<30\\ \frac{\pi }{3}, & \mathrm{for} & 30\leq t<40 \end{array}\right.$$

The weighting matrices of the objective function are heuristically selected as Q = diag(1.5, 1.5, 1.5) and R = diag(0.01, 0.01, 0.01). The parameters of both the DSTA and the DSMR were also heuristically tuned. To ensure a fair comparison, we used a fairly similar values as well as to prevent higher control signals which causes control signal saturation and chattering effect. The parameters of the proposed GA-DSTA, DSTA, and DSMR controllers are listed in Table 3.

To qualitatively evaluate the performance of the proposed controller, we used two performance indices: (i) the root mean square error (RMSE) was used to compute the tracking error of the three controllers for the surge, sway, and yaw rate; (ii) The Frobenius norm was used to obtain the total control energy expenditure of the three control algorithms. The RMSE and Frobenius norm are given by Equation (46) and Equation (47), respectively.

(46) $${★}_{\mathrm{RMSE}}=\sqrt{\frac{1}{N}\sum_{i=1}^{N}{\left({★}_{d}\left(k\right)-★\left(k\right)\right)}^{2}},$$

(47) $${∥U∥}_{F}=\sqrt{\sum_{i=1}^{M}\sum_{j=1}^{N}{\left|u_{ij}\right|}^{2}},$$

where ★ represents one of the three degrees of freedom, N is the number of time steps, and M denotes the number of degrees of freedom (that is, three in this case). U is a control signal matrix whose dimensions are the number of degrees of freedom by the number of time steps, and $u_{ij}$ denotes each element of U.

**Figure 6:**
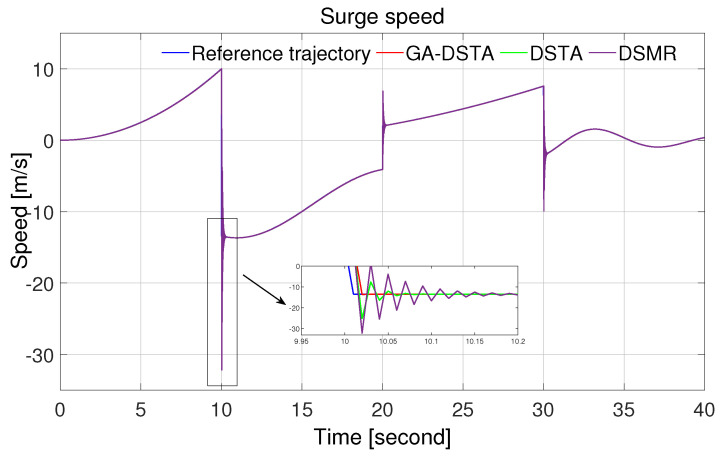
Surge speed trajectory following comparison of the GA-DSTA, DSTA, and DSMR controllers for Scenario 1.

**Figure 7:**
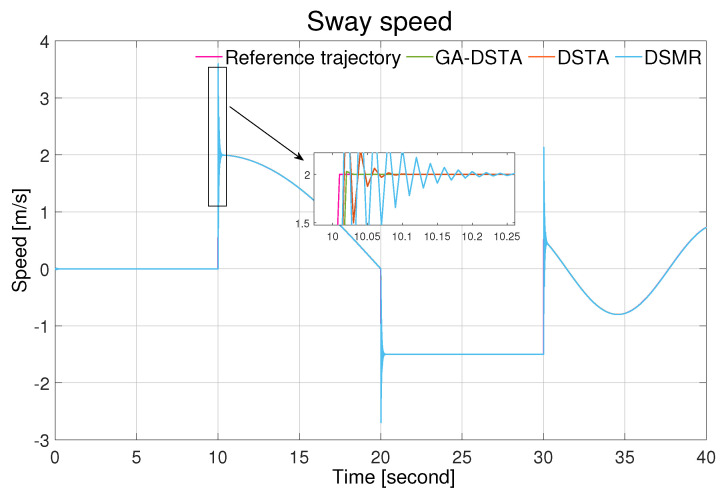
Sway speed trajectory following comparison of the GA-DSTA, DSTA, and DSMR controllers for Scenario 1.

**Figure 8:**
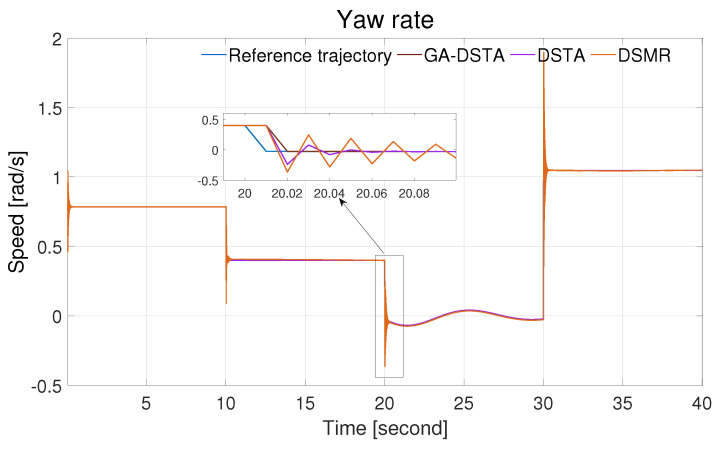
Yaw rate trajectory following comparison of the GA-DSTA, DSTA, and DSMR controllers for Scenario 1.

The simulation results of the three controllers for the nominal case of the surge, sway, and yaw rates are shown in Figure 6, Figure 7 and Figure 8. As can be observed from the simulation results, the three controllers drive the vehicle along the desired trajectory successfully; however, the proposed GA-DSTA controller outperforms the other two controllers both in terms of overshoot and tracking error minimization.

The RMSEs of the surge, sway, and yaw rate of the three controllers are listed in Table 4. It can be seen from this qualitative analysis that the proposed GA-DSTA controller yields a 13.5% and 40.01% reduction in RMSE compared to the DSTA and DSMR controllers, respectively, for the surge direction. Similarly, the percentage of reduction in RMSE was observed for the sway direction, except for the yaw rate of DSMR, which was approximately 42.56%.

**Figure 9:**
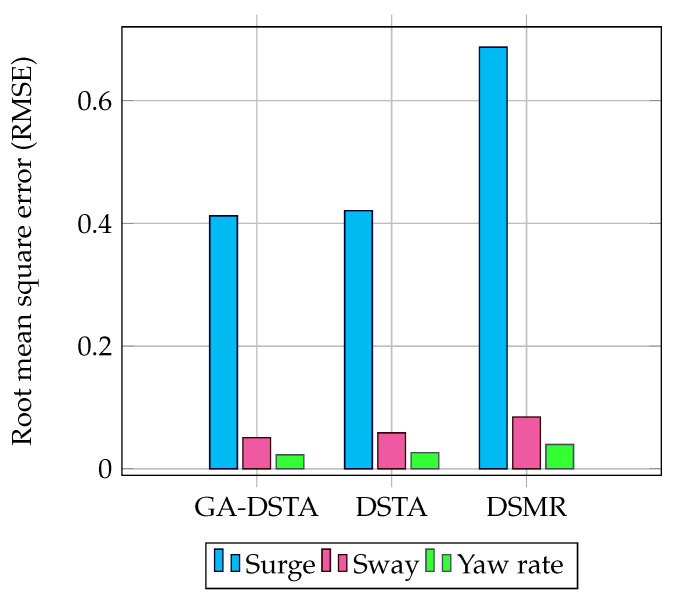
Comparison of RMSEs of GA–DSTA, DSTA, and DSMR controllers for surge, sway, and yaw rate for Scenario 1.

For the sake of brevity, the RMSEs of the three controllers are shown in the bar graph in Figure 9. As shown in the bar chart, the RMSE for the surge speed is higher than the RMSE for the sway speed and yaw rate for all the three control methods. This shows that when the speed increases, the tracking error increases as well.

Additionally, a step change was included in the desired trajectories to test the dynamic performance of the proposed controller. The proposed controller reacts quickly to the change and tracks the desired trajectory effectively, although there is an insignificant overshoot, as shown in Figure 6, Figure 7 and Figure 8. The conventional DSMR controller has a large overshoot compared to those of GA-DSTA and DSTA; however, it reacts immediately and settles almost within less than 0.2 s for the surge, sway, and yaw rate. This is due to the TDE algorithm, which estimates the unknown system dynamics so that the controller reacts quickly.

**Figure 10:**
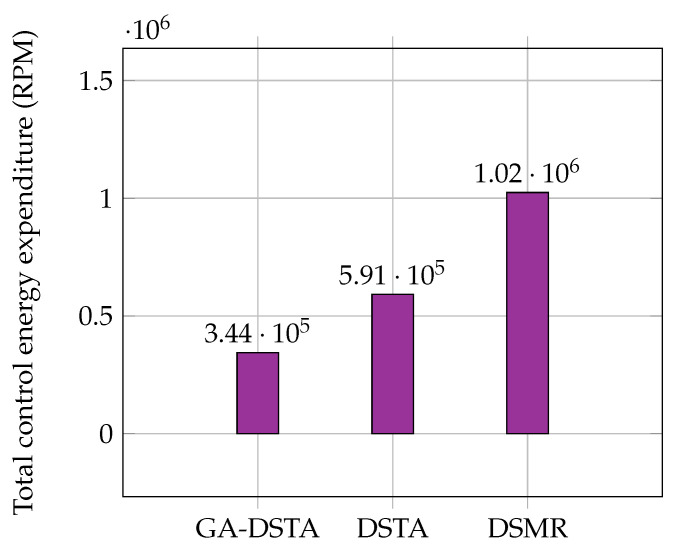
Comparison of the total energy expenditure of the GA–DSTA, DSTA, and DSMR controllers for Scenario 1. RPM is revolutions per minute.

The expenditure of control energy of the proposed GA-DSTA controller including those for DTSA and DSMR are given in Table 5. The energy consumption of the proposed controller was lower than those of the other two controllers. It was found that there was an 41.78% reduction in the energy expenditure of the GA-DSTA controller compared with that of the DSTA controller. However, the energy consumption of the GA-DSTA controller was reduced to 66.4% of that of the DSMR controller. This is clearly shown in the bar graph in Figure 10.

**Figure 11:**
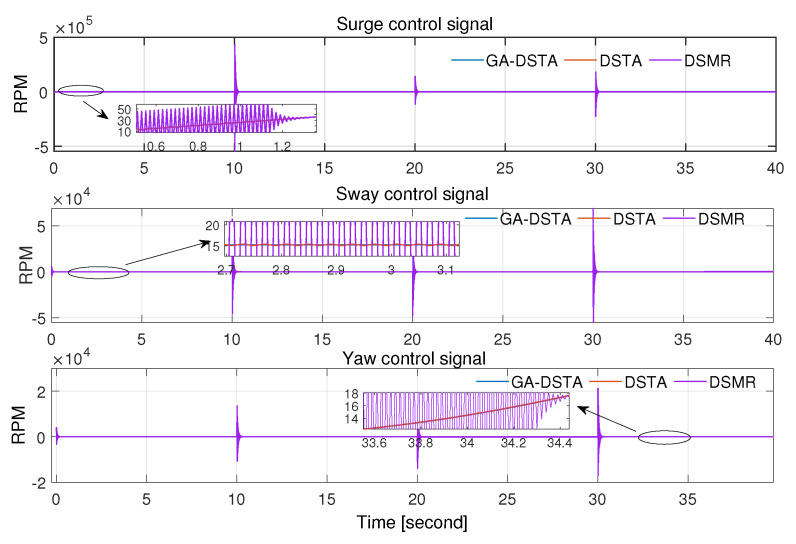
Control signals for surge, sway, and yaw rate of GA-DSTA, DSTA, and DSMR controllers for Scenario 1. RPM is revolutions per minute.

The total energy consumption of the proposed method and the other two controllers are listed in Table 5. As can be seen from this energy-consumption table, the proposed method has the lowest energy consumption. The energy consumption is related to the chattering. If the chattering is significant, then the energy consumption is high as well. The GA-DSTA controller significantly minimizes the chattering compared to both DSTA and DSMR controllers, resulting in the lowest energy consumption. Additionally, the chattering by the DSTA controller is lower than that of the conventional DSMR controller, leading to lower energy consumption as well. Despite the small difference in tracking errors across the three controllers, the notable improvement in chattering by the GA-DSTA and DSTA controllers contributes to the overall reduction in energy consumption. The chattering effect in the control signals for Scenario 1 is shown in Figure 11.

### 5.2. Scenario 2: External Disturbance

In the real world, the motion of the ASV is affected by external disturbances such as currents, wind, ocean waves, model uncertainties, and parametric variations. Thus, before deploying a control algorithm, its robustness against external disturbances must be tested. In this subsection, we present a simulation in the presence of external disturbances. Note that the external disturbances are assumed to be bounded and satisfy the Lipschitz condition given in Assumption 3. The external disturbances for the surge, sway, and yaw rates are given by Equations (48)–(50).

(48) $$u_{\mathrm{dis}}=\left\{\begin{array}{ccc} 0, & \mathrm{for} & 0\leq t<10\\ 1.5\sin(2\pi t)\cos(\pi t), & \mathrm{for} & 10\leq t<20\\ 1.5\mathrm{sawtooth}(2\pi t), & \mathrm{for} & 20\leq t<30\\ 0, & \mathrm{for} & 30\leq t<40 \end{array}\right.$$

(49) $$v_{\mathrm{dis}}=\left\{\begin{array}{ccc} 0, & \mathrm{for} & 0\leq t<10\\ 0.5\mathrm{sawtooth}(0.5\pi t), & \mathrm{for} & 10\leq t<20\\ 0.5\sin(0.2\pi t)\cos(0.2\pi t), & \mathrm{for} & 20\leq t<30\\ 0, & \mathrm{for} & 30\leq t<40 \end{array}\right.$$

(50) $$r_{\mathrm{dis}}=\left\{\begin{array}{ccc} 0.2\sin(0.5\pi t)\cos(0.4t)\\ -0.1\sin(0.2t), & \mathrm{for} & 0\leq t<10\\ 0, & \mathrm{for} & 10\leq t<20\\ 0.2\mathrm{sawtooth}(0.5\pi t), & \mathrm{for} & 20\leq t<30\\ 0, & \mathrm{for} & 30\leq t<40 \end{array}\right.$$

where sawtooth is a MATLAB built-in sawtooth function. For the surge and sway directions, disturbances were added to the reference trajectory from *t* = 10 to *t* = 30, and disturbances for the yaw rate were added from *t* = 0 to *t* = 10 and *t* = 20 to *t* = 30. The maximum magnitudes of the disturbances for the surge $u_{\mathrm{dis}}$, sway $v_{\mathrm{dis}}$, and yaw rate $r_{\mathrm{dis}}$ were ±1.5 m/s, ± 0.5 m/s, and ± 0.2 rad/s, respectively. The sampling time was the same as that in the nominal case.

**Figure 12:**
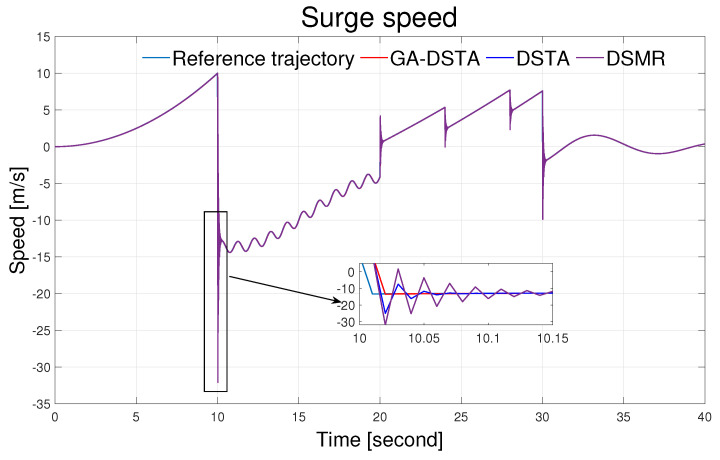
Surge speed trajectory following comparison of the GA-DSTA, DSTA, and DSMR controllers for Scenario 2.

**Figure 13:**
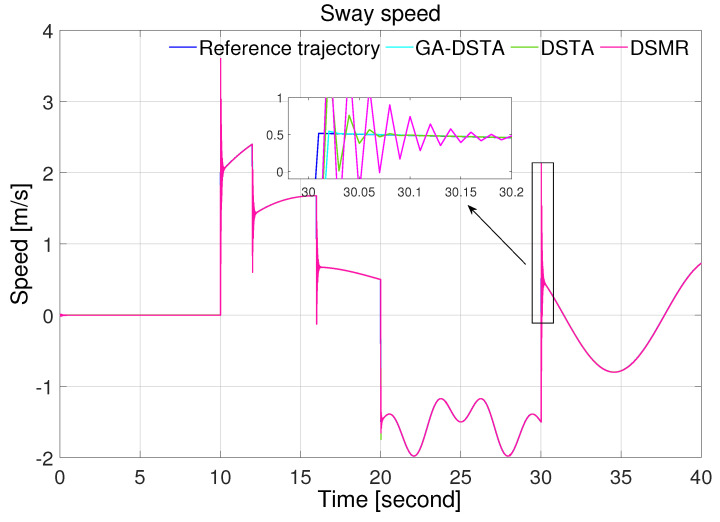
Sway speed trajectory following comparison of the GA-DSTA, DSTA, and DSMR controllers for Scenario 2.

**Figure 14:**
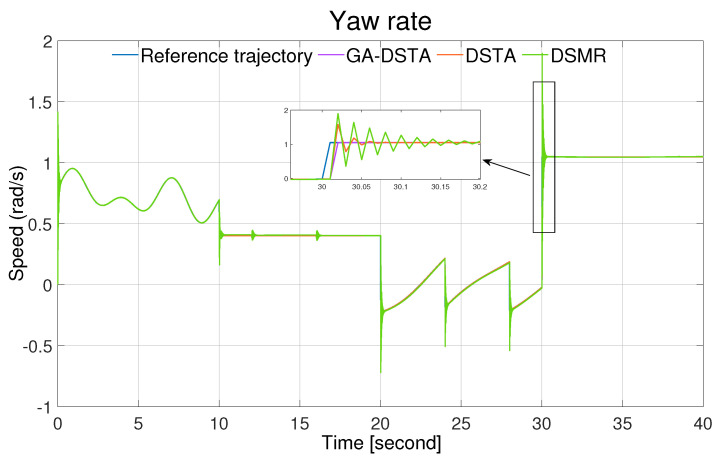
Yaw rate trajectory following comparison of the GA-DSTA, DSTA, and DSMR controllers for Scenario 2.

The simulation results for the second scenario are shown in Figure 12, Figure 13 and Figure 14 for the surge, sway, and yaw rates, respectively. Even in the presence of external disturbances, the proposed method, including the other two controllers, successfully followed the desired trajectory.

**Figure 15:**
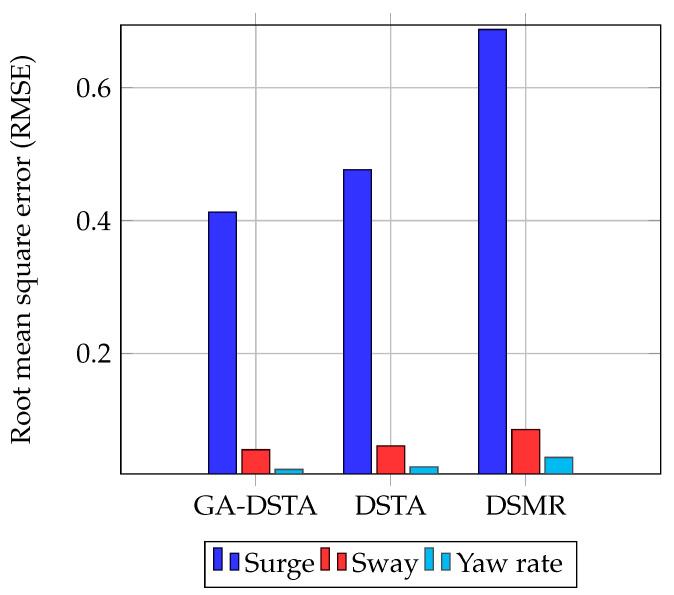
Comparison of the RMSEs of the GA-DSTA, DSTA, and DSMR controllers for the surge, sway, and yaw rate Scenario 2. RPM is revolutions per minute.

The RMSEs for the second scenario are given in Table 6. The percentages of RMSEs reduction of the proposed controller compared to the DSTA controller for the surge, sway direction, and yaw rate are approximately 13.39%, 9.046%, and 13.36%, respectively, and 40%, 35.44%, and 41.7% as compared to the DSMR, respectively. From this comparison, it can be inferred that the proposed controller outperforms the other two. The RMSEs are depicted in the bar chart in Figure 15.

The robustness of the proposed controller to external disturbances relies on the gains of the controllers $\Omega_{1}$ and $\Omega_{2}$. When the values of these gains are small, the chattering effect decreases, and the robustness of the controller degrades. The GA tunes these gains to smaller values based on a given objective function. This slightly could degrade the robustness of the proposed controller. However, the presence of the TDE algorithm prevents further performance degradation by estimating unknown system dynamics and external disturbances.

**Figure 16:**
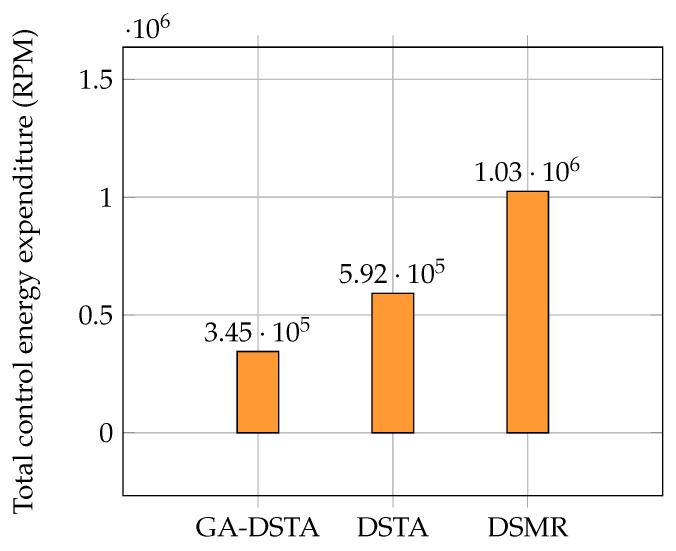
Comparison of total energy expenditure of the GA-DSTA, DSTA, and DSMR controllers in revolution for Scenario 2. RPM is revolutions per minute.

**Figure 17:**
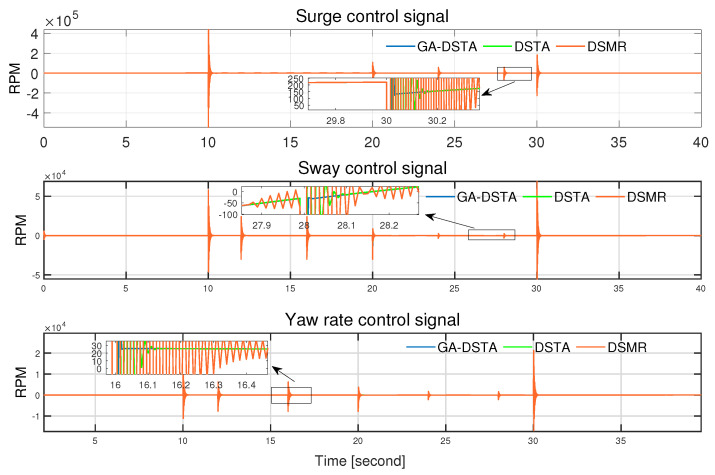
Control signals for surge, sway, and yaw rate of GA-DSTA, DSTA, and DSMR controllers for Scenario 2. RPM is revolutions per minute.

Regarding the control energy expenditure, the proposed controller utilized lower energy than the DSTA and DSMR controllers as shown in Table 7. There is a 41.6% reduction in the energy consumption of GA-DSTA compared to that in DSTA, and 66.3% compared to that in DSMR. The bar chart in Figure 16 shows the total energy consumed by the three controllers. The proposed GA-DSTA and DSTA controllers have less chattering compared to DSMR controller as shown in Figure 17.

## 6. Conclusions

In this study, a GA-DSTA controller was proposed for trajectory following of an ASV. Besides, a TDE algorithm was incorporated to estimate the unknown dynamics of the system. The stability of the closed-loop system was analyzed using a Lyapunov approach. The controller gains were tuned using GA based on a quadratic performance index, the sum of the tracking error and the control energy. The performance of the proposed controller was validated with and without the presence of external disturbances, and its performance was also compared with both DSTA and DSMR. The results showed the proposed GA-DSTA controller outperformed, in terms of both the tracking error and the chattering phenomenon reduction, the other controllers. However, though GA improved the tracking capability of the proposed method by suppressing the chattering effect, its robustness slightly decreased when external disturbances were added. However, the TDE algorithms improved the robustness of the controller.

The proposed controller can be applied to the trajectory following of an ASV which can be used to replace time-consuming, expensive, and hazardous sensing tasks which were previously performed by manned marine vehicles. In the future, the practical implementation of the proposed controller will be done. 

## Figures and Tables

**Table 1:** Parameter setting of genetic algorithm.

Parameter	Value
Population size	250
Maximum generation	15
stall generation	7
Crossover	Two point cross
Selection function	Roulette
Elite count	2
Crossover fraction	0.8
Mutation rate	0.01
Mutation function	Uniform
Migration direction	Forward

**Table 2:** Physical parameters of the ASV.

Parameter	Value	Unit	Parameter	Value	Unit
$X_{\dot{u}}$	−3.5	kg · m	$X_{|u|u}$	−6.822	kg · s/m
$Y_{\dot{v}}$	−41.55	kg · m	$Y_{|v|v}$	−71	kg · s/m
$N_{\dot{v}}$ = $Y_{\dot{r}}$	−14	kg · m	$Y_{|r|r}$	−35.52	kg/s
$N_{\dot{r}}$	−28	kg · m^2^	$N_{|v|v}$	20.6	kg/s
$X_{u}$	−4.27	kg/s	$N_{|r|r}$	−11.52	kg · m^2^ · s
$Y_{v}$	−25.63	kg/s	*m*	125.37	kg
$Y_{r}$ = $N_{v}$	−19.44	kg · m/s	$x_{g}$	0.232	m
$N_{r}$	−32.65	kg · m^2^ · s	$I_{z}$	1.5	kg · m

kg: kilogram, s: second, and m: meter

**Table 3:** GA-DSTA, DSTA, and DSMR controllers’ gain setting.

Controller	Parameter	Value
	$K_{p}$	diag(0.0023, 0.1006, 0.3481)
	$K_{1}$	diag(0.0081, 0.0145, 0.0037)
GA-DSTA	$K_{2}$	diag(0.8750, 0.8628, 0.3212)
	$\Omega_{1}$	diag(0.0009, 0.0008, 0.0006)
	$\Omega_{2}$	diag(0.0009, 0.0003, 0.0008)
	$K_{p}$	diag(0.4, 0.4, 0.4)
	$K_{1}$	diag(0.5, 0.5, 0.5)
DSTA	$K_{2}$	diag(0.3, 0.3, 0.3)
	$\Omega_{1}$	diag(0.02, 0.02, 0.02)
	$\Omega_{2}$	diag(0.02, 0.02, 0.01)
	$K_{p}$	diag(0.8, 0.8, 0.8)
DSMR	q	diag(20, 20, 20)
	ϵ	diag(0.02, 0.02, 0.02)

**Table 4:** RMSEs of GA-DSTA, DSTA, and DSMR controllers for surge, sway, and yaw rate for Scenario 1.

GA-DSTA			DSTA			DSMR		
$u_{\mathrm{RMSE}}$	$v_{\mathrm{RMSE}}$	$r_{\mathrm{RMSE}}$	$u_{\mathrm{RMSE}}$	$v_{\mathrm{RMSE}}$	$r_{\mathrm{RMSE}}$	$u_{\mathrm{RMSE}}$	$v_{\mathrm{RMSE}}$	$r_{\mathrm{RMSE}}$
0.4123	0.0508	0.0228	0.4761	0.0586	0.0264	0.6873	0.0845	0.0397

**Table 5:** The total energy expenditure of GA-DSTA, DSTA, and DSMR controllers for surge, sway, and yaw rate for Scenario 1. RPM is revolutions per minute.

GA-DSTA	DSTA	DSMR
${∥U∥}_{F}$ (RPM)	${∥U∥}_{F}$ (RPM)	${∥U∥}_{F}$ (RPM)
$3.4419\times 10^{5}$	$5.9119\times 10^{5}$	$1.0244\times 10^{6}$

**Table 6:** RMSEs of the GA-DSTA, DSTA, and DSMR controllers for the surge, sway, and yaw rate for Scenario 2.

GA-DSTA			DSTA			DSMR		
$u_{\mathrm{RMSE}}$	$v_{\mathrm{RMSE}}$	$r_{\mathrm{RMSE}}$	$u_{\mathrm{RMSE}}$	$v_{\mathrm{RMSE}}$	$r_{\mathrm{RMSE}}$	$u_{\mathrm{RMSE}}$	$v_{\mathrm{RMSE}}$	$r_{\mathrm{RMSE}}$
0.4126	0.0550	0.0253	0.4764	0.0608	0.0292	0.6877	0.0852	0.0434

**Table 7:** The total energy expenditure of the GA-DSTA, DSTA, and DSMR controllers for the surge, sway, and yaw rate for Scenario 2. RPM is revolutions per minute.

GA-DSTA	DSTA	DSMR
${∥U∥}_{F}$ (RPM)	${∥U∥}_{F}$ (RPM)	${∥U∥}_{F}$ (RPM)
$3.4524\times 10^{5}$	$5.9207\times 10^{5}$	$1.0250\times 10^{6}$

## Data Availability

Data is contained within the article.

## Appendix A. Proof of Equation (33)

$$\begin{array}{ll} \varphi^{T}F\varphi & ={\left[\begin{array}{c} -T\Omega_{1i}{\left|s_{i}\right|}^{\frac{1}{2}}\\ -T^{2}\Omega_{2i}sgn\left(s_{i}\left(k\right)\right) \end{array}\right]}^{T}\left[\begin{array}{cc} f_{11} & f_{12}\\ f_{21} & f_{22} \end{array}\right]\left[\begin{array}{c} -T\Omega_{1i}{\left|s_{i}\right|}^{\frac{1}{2}}\\ -T^{2}\Omega_{2i}sgn\left(s_{i}\left(k\right)\right) \end{array}\right]\\ & =T^{2}\Omega_{1i}^{2}f_{11}|s_{i}\left(k\right)|+2T^{3}f_{12}\Omega_{1i}\Omega_{2i}{\left|s_{i}\left(k\right)\right|}^{\frac{1}{2}}+T^{4}f_{22}\Omega_{2i}^{2}\\ & =(\varrho_{1}|s_{i}\left(k\right)|+\varrho_{2})|s_{i}{\left(k\right)|}^{\frac{1}{2}}+\varrho_{3}\\ & \leq \varrho_{1}|s_{i}\left(k\right)|+\varrho_{2}{\left|s_{i}\left(k\right)\right|}^{\frac{1}{2}}+\varrho_{2}+\varrho_{3}\\ & \leq \left(\varrho_{1}+\varrho_{2}\right)\left|s_{i}\left(k\right)\right|+\varrho_{2}+\varrho_{3}\\ & =\bar{\varrho_{1}}\left|s_{i}\left(k\right)\right|+\bar{\varrho_{2}}, \end{array}$$

where $\varrho_{1}=T^{2}\Omega_{1i}^{2}f_{11}$, $\varrho_{1}=2T^{3}f_{12}\Omega_{1i}\Omega_{2i}$, $\varrho_{3}=T^{4}f_{22}\Omega_{2i}^{2}$, $\bar{\varrho_{1}}=\varrho_{1}+\varrho_{2}$, and $\bar{\varrho_{2}}=\varrho_{2}+\varrho_{3}$, F is a positive definite matrix. And also, $\Omega_{1i}>0$ and $\Omega_{2i}>0$.
